# Abscesses of Both Kidneys Due to Chronic Cystitis, Abscesses Opened and Drainage Kept up, Steady Improvement*Reported in part to Richmond Academy of Medicine and Surgery, May 11th, 1897, and notes up to May 25th added.

**Published:** 1897-07

**Authors:** Jacob Michaux

**Affiliations:** Richmond, Va.; Professor of Obstetrics, University College of Medicine, Richmond, Va., etc.; 323 East Franklin Street


### Selections and Abstracts.

### ABSCESSES OF BOTH KIDNEYS DUE TO CHRONIC CYSTITIS.—ABSCESSES OPENED AND DRAINAGE KEPT UP.—STEADY IMPROVEMENT.*

By JACOB MICHAUX, M. D., Richmond, Va.

Professor of Obstetrics, University College of Medicine, Richmond, Va., etc.

I desire to report a case of chronic cystitis, of five or six years' standing, resulting in abscess of the kidney, and the surgical measures resorted to for relief.

Miss C. F., white, æt. 22, was brought to me in January, 1897. She was found to be suffering with chronic cystitis, which had been present, according to her statement and that of her aunt, for five or six years. I advised operative treatment, but could not then induce her to submit. I put her upon medical treatment, and advised rest. After the lapse of a month of fruitless effort, she consented to go to the Retreat for the Sick of this city for operation.

Accordingly, on the 18th of February, 1897, I dilated the urethra under ether and cauterized the internal surface of the bladder. She was then put to bed, and the bladder washed out daily with a solution of teaspoonful of boric acid to a pint of tepid water. The urine drained away well, and the temperature and general condition improved fairly well for about three weeks.

About 15th of March, the temperature again began to climb until the 24th of March, when it reached 104° F.

For some days my attention had been attracted to the peculiar character and location of the pain, which did not seem to come, as I at first supposed, from the bladder, but was referred to the left side radiating outward and upward *through* the *left* inguinal region and over the crest of the ilium to the region of the kidney. The post-renal region was not enlarged, but was very tender. The bladder symptoms were at this period in abeyance, and yet we had fever and pus in the urine in large (visible) quantities. I, therefore, reasoned that it came from the kidney. In order to confirm the diagnosis, I sent a vial of the urine to Dr. Moses D. Hoge, Jr., Professor of Urinology and Pathology in the University College of Medicine, who, upon a careful and most complete analysis, reported renal cells among the debris.

---

* Reported in part to Richmond Academy of Medicine and Surgery, May 11th, 1897, and notes up to May 25th added.

"Upon this hint I spake," and operated the following day (March 25th, 1897).

After exposing the whole posterior surface of the kidney I saw that the entire organ was enlarged and inflamed. The pelvis of the kidney did not (as I expected it would) show any signs of distension and inflammation.

Dr. J. W. Henson, Professor of Anatomy in the University College of Medicine, who assisted me, called my attention, at this juncture, to a peculiar yellowish spot near the convex border, not far from the middle of the organ. On palpation, this was found to be the thin wall of a large abscess wholly within the organ. This was incised, and my index finger was carried down to the pelvis of the kidney, and found an abscess cavity about an inch and a half wide, extending the whole width of the organ, and at that point almost dividing the kidney into two parts, the capsule being about the only tissue that held the separated portions together. I was tempted to remove the whole kidney—so unpromising was the condition presented; but concluded to try to save whatever sound tissue might remain by inserting a rubber drainage tube into the kidney abscess, and dressing the wound by the open method. I put two stitches in the posterior end of the long incision and packed the rest with gauze around the tube. Drs. Wm. T. and St. J. Oppenheimer, J. A. Hodges, J. W. Henson, Wm. R. Jones, J. M. Winfree, also Drs. Porter and Greiner, house physicians in the Retreat, kindly assisted me.

The temperature at once fell to normal, and, with trifling variations, remained there. The general condition improved, and there was strong reason to hope for a good recovery.

About the 1st of April, however, I was disappointed to find more temperature and bladder symptoms looming up. I combatted these as best I could by drug treatment and lavage, but without success, till April 15th, when I opened the bladder between the mouths of the ureters, and inserted a glass drainage tube about five-eighths inch in diameter. This was followed by a great improvement in temperature and general condition, and greatly facilitated washing out and drainage of the viscus. I soon began to take her out of bed and make her sit in a rocker and take a few steps. [The condition of the kidneys continued to improve, save on one occasion, when I removed the tube, about two weeks after the operation upon it. Finding the temperature going up, I reinserted it, and it still remains, draining limpid urine and only small amounts of pus.] The bladder is now (May 12th) giving her more trouble than the kidney, and I fear that its walls, so long inflamed, may not be able to take on reparative action.

I shall now try continuous irrigation for three or four hours night and morning.

Just after the above report, I found that although the tube from the left kidney was draining well, the one from the bladder was dry, and that there was *pain* and *tenderness* in the *right* inguinal and lumbar regions, clearly showing stoppage of the right ureter. I therefore, on the 14th of May (1897)—thirty hours after these symptoms first appeared—operated under ether upon the right kindey in order to relieve the choked ureter.

The incision was made just below (three-fourths of an inch) the lower border of the last rib. The kidney was exposed, and the pelvis and upper end of the ureter were brought into view. An incision was made at the junction of the ureter and pelvis, and the urine issued forth in a strong jet some six inches above the body of the patient. The quantity of the urine that escaped was considerable, and some clots and a little pus escaped. The depression and nausea following this operation were extreme, and I almost despaired of her life; but she rallied finally and is now improving, though still weak. The drainage from both kidneys still continues through the rubber drainage-tubes in the incisions in the back, and there is some urine from the glass drainage-tubes in the base of the bladder.

I shall keep the kidneys and bladder well drained and washed out, with the hope that healing may ultimately take place, and that then the three fistulæ, which I have made, as above described, may be closed. She is free from pain now, for the first time in many months, and is slowly gaining strength, but I fear for her yet.

*323 East Franklin Street.*

